# Relationship of red splenic arteriolar hyaline with rapid death: a clinicopathological study of 82 autopsy cases

**DOI:** 10.1186/1746-1596-7-182

**Published:** 2012-12-31

**Authors:** Hirokazu Kotani, Masashi Miyao, Sho Manabe, Tokiko Ishida, Chihiro Kawai, Hitoshi Abiru, Keiji Tamaki

**Affiliations:** 1 Department of Forensic Medicine and Molecular Pathology, Kyoto University Graduate School of Medicine, Yoshida-Konoe-cho, Sakyo-ku, Kyoto, 606-8501, Japan

**Keywords:** Splenic arteriolar hyaline, Tinctorial pattern, Azan stain, Cause of death, Rapidity of death

## Abstract

**Background:**

Little is known about the relationship between splenic arteriolar hyaline and cause of death. The purpose of this retrospective study was to evaluate the clinicopathological significance of splenic arteriolar hyaline in autopsy cases and estimate the applicability of hyaline for diagnosing the cause and rapidity of death.

**Methods:**

Archival data and histological slides from 82 cases were reviewed retrospectively. One section of each spleen was evaluated microscopically. The tinctorial pattern of splenic arteriolar hyaline was examined with Heidenhain’s Azan trichrome stain, and the relationships between this pattern and age, cause of death, and rapidity of death were investigated.

**Results:**

Fifty-four cases demonstrated hyaline change, with 3 different tinctorial patterns: red, blue, and a combination of red and blue. The 3 patterns coexisted in various proportions in each tissue section. Frequency of the blue pattern increased with age (*P* < 0.01) and was unrelated to cause of death. By contrast, the red pattern was unrelated to age and appeared with different frequency according to cause of death. The red pattern appeared with significantly higher frequency in the circulatory disease group and the drowning and asphyxia group (both *P* < 0.01). Moreover, the presence of the red pattern had high specificity for the detection of rapidly fatal cases. The combination of the 2 colors was excluded from clinicopathological analyses due to its admixed nature.

**Conclusions:**

Estimation of splenic arteriolar hyaline with Heidenhain’s Azan trichrome stain is useful for assessment of the cause and rapidity of death.

**Virtual slides:**

The virtual slide(s) for this article can be found here: http://www.diagnosticpathology.diagnomx.eu/vs/1132441651796836

## Background

Hyaline arteriolosclerosis is one of the most common degenerative changes that occurs throughout the body and is found frequently in the spleen as well as the kidney [1,2]. Many investigators have described clinicopathological characteristics of splenic arteriolar hyaline [3-8]. This change has been demonstrated to increase in frequency with age and result from a general process that is likely hemodynamic, e.g., hypertension [2,6,9-12]. However, little attention has been given to the relationship between arteriolar hyaline and cause of death.

Hyaline in an artery or arteriole appears as a pink, amorphous thickening with associated luminal narrowing in routine histologic sections with hematoxylin and eosin stain [2] and contains the following major components: fibrin, immunoglobulin, complement, and damaged basement membranes [1,7,12-17]. Hyaline arteriosclerosis develops in incremental steps and demonstrates different properties at each stage of the hyaline aging process [11,18]. Heidenhain’s Azan trichrome stain (Azan stain) shows tinctorial variety according to the properties of the different stages: fresh hyaline is red, and old hyaline is blue [18-21]. Although this property of the Azan stain appears useful for investigating the pathological effects of hyaline change in a variety of diseases, few studies have been conducted using this property. Feigin et al. used these tinctorial properties to investigate the clinicopathological characteristics of the cerebral arteries in autopsy cases and reported that the red pattern (fresh hyaline) is frequently observed in cerebral hemorrhage [18]. However, little information is available beyond this study concerning the clinicopathological assessment of the tinctorial varieties of hyaline change.

Information concerning the rapidity of death, e.g., sudden death or not, provides one of the most valuable clues for diagnosing the cause of death in forensic autopsy. However, rapidity must be determined by information from witnesses in most practical cases because no pathological findings have been established to indicate sudden death *per se*[22-25]. Therefore, the identification of a common histological feature shared in cases of sudden death from all causes would be helpful for pathologists in autopsy practice. The purpose of this retrospective study was to evaluate the clinicopathological significance of splenic arteriolar hyaline in autopsy cases and estimate the applicability of hyaline for diagnosing the cause and rapidity of death.

## Cases and methods

### Case selection

We retrieved case documents and microscopic slides of the spleen from the archives of the Department of Forensic Medicine and Molecular Pathology, Kyoto University Graduate School of Medicine, Japan, from October 2009 to April 2011. The cases were selected based on the availability of archival slides rather than consecutive autopsies. A total of 157 cases were available, of which 88 had microscopic slides of the spleen. Six cases were excluded due to inappropriate preparation of the slides. Characteristics of the remaining 82 cases are shown in Table 1.

**Table 1:** Characteristics of study groups

	**All cases**	**Rapidly fatal cases**	**Presumed cases**	**Non-rapidly fatal cases**	**Unknown cause cases**
**Sample size**	82	26	17	35	4
**Man/Woman**	52/30	18/8	11/6	21/14	2/2
**Age groups**					
≤ 19 years	14	6	4	3	1
20-39 years	17	2	7	7	1
40-59 years	18	8	2	8	0
60-79 years	28	10	4	13	1
80 years ≤	5	0	0	4	1
Mean age ± SD	47 ± 25	46 ± 24	36 ± 24	53 ± 25	47 ± 39
**Median PMI (hr)**	41.0	35.5	30.0	47.0	35.5
**Causes of death**					
Traumas	18	9	3	6	-
Circulatory diseases	15	6	9	0	-
Other internal diseases	14	1	3	10	-
Hypo-, Hyperthermia	14	1	0	13	-
Drowning & Asphyxia	11	9	2	0	-
Intoxication	6	0	0	6	-
Unknown	4	-	-	-	-

*SD*, indicates standard deviation; *PMI*, postmortem interval.

Fifty-two patients (63.4%) were men, and 30 (37.6%) were women. The cases were divided into 4 categories: rapidly fatal cases, presumed rapidly fatal cases, non-rapidly fatal cases, and cases with unknown cause of death. Twenty-six cases were categorized as rapidly fatal cases that progressed to death less than 1 hour from the onset of the fatal event. Rapidity was determined based on the presence of a credible witness or document. Possible cases of rapid progression without credible evidence (n = 17) were categorized as presumed rapidly fatal cases instead of rapidly fatal cases. The remaining cases were categorized as non-rapidly fatal cases (n = 35). The cases with unknown cause of death were excluded from statistical analyses (n = 4).

The cases were also divided into 5 groups according to age and into 7 groups according to cause of death. The trauma group consisted of hemorrhagic shock (n = 10), head injury (n = 7), and thoracic injury (n = 1); the circulatory disease group consisted of coronary artery disease (n = 7), non-ischemic heart disease (n = 6), and stroke (n = 2); the other internal disease group consisted of infection (n = 10) and gastrointestinal disease (n = 4); the hypo- and hyperthermia group included 14 cases, 8 hypothermia and 6 hyperthermia; and the drowning and asphyxia group included 11 cases, 6 drowning and 5 asphyxia. The cause of death was concluded by forensic pathologists (H.K. and K.T.) based on overall evidence composed of the course of events and circumstances, external and internal autopsy findings, microscopic examinations, and toxicological and biochemical analyses, as well as the exclusion of other causes of death. This study was conducted within the framework of the Ethics Committee of Kyoto University.

### Histopathology

A single histological section from each spleen was examined. The tissues were fixed in 10% neutral buffered formalin for approximately 1 month, embedded in paraffin, serially cut at 3 μm, and routinely stained with hematoxylin and eosin stain (HE stain) and Azan stain [20,21]; vessel hyaline was assessed throughout the entire section. Hyaline was defined microscopically as a homogeneous, translucent, eosinophilic thickening of the intima on HE stain [2]. Central arteries and penicillar arterioles were examined, but trabecular arteries and capillaries were not [26,27]. Sections with one or more affected vessels were considered positive. We referred to the hyaline observed in those vessels as splenic arteriolar hyaline in this study. Although the tunica media and the elastic lamina of the vessels showed variable staining with Azan, their appearance was not taken into account. The assessment of hyaline was performed with a blind review of the cases by a pathologist (H.K.).

### Statistical analyses

The statistical relationships between the positive ratio of splenic arteriolar hyaline and age, cause of death, and rapidly fatal cases were analyzed with analysis of variance. The Cochran-Armitage test and logistic regression model were used to assess trends in age. Ryan’s multiple comparison test was used for analysis of the relationship with cause of death, and the *χ*^2^ contingency test was used to determine the relationship with rapidly fatal cases. *P* values less than 0.05 were considered statistically significant. The diagnostic accuracy of hyaline for rapidly fatal cases was estimated based on sensitivity, specificity, positive predictive value, and negative predictive value. All statistical analyses were performed using R software for Windows, version 2.14.1.

## Results

### Tinctorial patterns of splenic arteriolar hyaline and their frequencies of appearance

**Figure 1:**
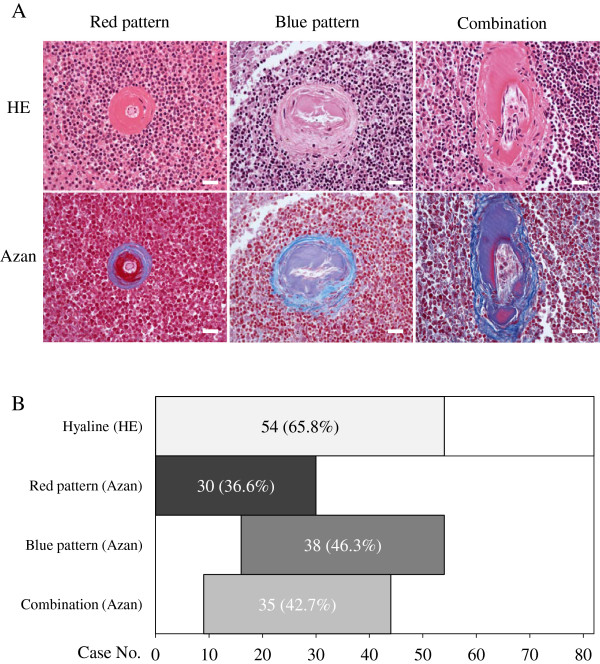
**Characteristics of splenic arteriolar hyaline with Azan stain. A**: Representative images of 3 tinctorial patterns (upper panels, HE stain; lower panels, Azan stain; scale bars: 50 μm). *Similar hyaline depositions on HE stain* present 3 different staining patterns with Azan stain: red, blue, and a combination of the two. **B**: The number of cases demonstrating each tinctorial pattern among 82 forensic autopsy cases. The 3 patterns coexisted in various proportions in each case.

Hyaline changes in splenic arterioles on HE staining appeared as amorphous, translucent, eosinophilic thickening of the intimas as shown in the upper panels of Figure 1A. Meanwhile, Azan staining turned each hyaline 1 of the 3 following tinctorial patterns, as shown in the lower panels: red, blue, and a combination of the 2 colors. These hyaline changes affected either the entire circumference of a vessel at a given level or only a part of the circumference; both were counted as positive vessels. We investigated the appearance frequencies of each tinctorial pattern in all 82 cases prior to the evaluation of clinicopathological characteristics (Figure 1B). Fifty-four (65.8%) cases demonstrated 1 or more positive vessel. Of these vessels, the red pattern was found in 30 (36.6%) cases, the blue pattern in 38 (46.3%), and the combination in 35 (42.7%). The 3 patterns coexisted in the following proportions in each tissue section: all 3 patterns in 15 (18.3%) cases and 2 patterns in 20 (24.3%). Meanwhile, some cases demonstrated only 1 pattern: 9 (11.0%) cases with the red and 10 (12.2%) with the blue pattern. No case demonstrated only the combination pattern, which was excluded from the following clinicopathological analyses due to its admixed nature.

We compared postmortem intervals (PMIs) between the cases with the only blue pattern and only red pattern to evaluate whether PMI leads to changes in staining patterns. The PMI of cases with the only blue pattern ranged from 11 to 240 h, with a mean interval of 63.7 h. The only red pattern was found in cases with PMIs ranging from 28 to 192 h, with mean interval of 58.6 h. In addition, PMIs of cases with both staining patterns in each tissue section ranged from 17 to 240 h. These results indicate that PMI did not lead to changes in staining patterns.

### The relationship of age with the blue and red patterns

**Figure 2:**
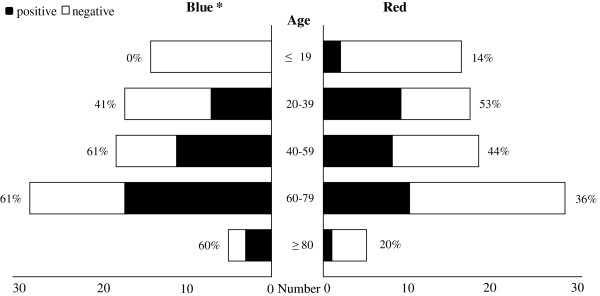
**The number and frequency of cases demonstrating the blue and red pattern in each age group.** The black bar indicates the number of positive cases; white bar indicates negative cases. The positive ratios of blue and red pattern in each group are described at the left and right side of each bar, respectively. The blue pattern was increasingly frequent with age (* *P* < 0.01, Cochran-Armitage test and logistic regression model), while the red pattern was unrelated to age.

We investigated the appearance frequencies of the blue and red patterns in groups stratified by age, as previous studies have reported that the incidence of hyaline change increases with age [3-5,28]. The results are summarized in Figure 2. Thirty-eight cases demonstrated the blue pattern, including 25 men and 13 women, with a male to female ratio of 1.92:1. Cases ranged from 29 to 91 years old, with mean and median ages of 57.1 and 60.0 years, respectively. The blue pattern was not found in the younger age group and was increasingly frequent with age, as in previous studies (Figure 2, left column) (*P* < 0.01, Cochran-Armitage test and logistic regression model). Meanwhile, 30 cases demonstrated the red pattern, including 22 men and 8 women, with a male to female ratio of 2.75:1. This pattern was found in cases ranging from 18 to 90 years old, with mean and median ages of 50.0 and 52.5 years, respectively. Unlike the blue pattern, the appearance frequencies did not significantly differ across age groups (Figure 2, right column) (*P* = 0.71, Cochran-Armitage test and logistic regression model).

### The relationship of cause of death with the blue and red patterns

**Figure 3:**
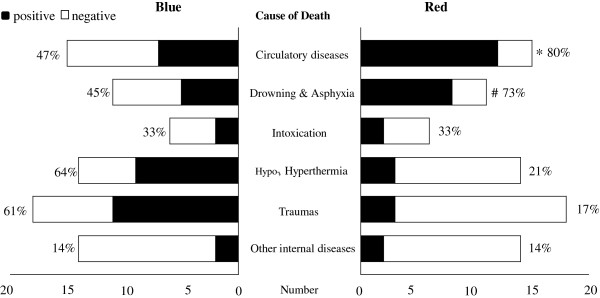
**The number and frequency of cases demonstrating the blue and red pattern in each cause-of-death group.** The black bar indicates the number of positive cases; white bar indicates negative cases. The positive ratios of blue and red pattern in each group are described at the left and right side of each bar, respectively. The red pattern appeared with significantly higher frequency in the circulatory diseases and drowning and asphyxia groups, and with low frequency in the remaining 4 groups (*P < 0.01 vs. hypo- and hyperthermia, trauma, and other internal diseases; #*P <* 0.01 vs. trauma and other internal diseases; Ryan’s multiple comparison test). By contrast, the blue pattern was unrelated to cause of death.

Next, we investigated the appearance frequencies of the blue and red patterns in each group stratified by cause of death to elucidate the applicability of these patterns to the diagnosis of cause of death in forensic autopsy. The results are summarized in Figure 3.

The relationship of the blue pattern to causes of death is shown in Figure 3, left column. This pattern was found in all causes of death: hypo- and hyperthermia (64%, 9/14), trauma (61%, 11/18), circulatory disease (47%, 7/15), drowning and asphyxia (45%, 5/11), intoxication (33%, 2/6), and other internal diseases (14%, 2/14), in order of descending frequency. Ryan’s multiple comparison test did not indicate a significant difference in appearance frequencies between any 2 cause-of-death groups.

The relationship of red pattern to causes of death is shown in Figure 3, right column. Unlike the blue pattern, this pattern exhibited different frequencies of appearance across the cause of death groups. The groups were classified into 2 categories according to the high or low appearance frequency of the red pattern. The category with high frequency of the red pattern contained 2 cause-of-death groups: circulatory disease (80%) and drowning and asphyxia (73%). The category with low frequency of the red pattern consisted of the remaining 4 groups: intoxication (33%, 2/6), hypo- and hyperthermia (21%, 3/14), trauma (17%, 3/18), and other internal diseases (14%, 2/14). Ryan’s multiple comparison test revealed that the positive ratio of the circulatory disease group was significantly higher than those of the hypo- and hyperthermia, trauma, and other internal disease groups (*P* < 0.01), and the positive ratio of the drowning and asphyxia group was also significantly higher than those of the hypo- and hyperthermia group and the other internal disease group (*P* < 0.01).

Even in the 4 groups demonstrating a low positive ratio of the red pattern, 10 positive cases were observed. Half of them were categorized as rapidly or presumed rapidly fatal cases: one 18-year-old athlete due to hyperthermia, 3 trauma cases due to falling without fatal bleeding, and one 35-year old man with myocarditis. The other 5 positive cases in these groups were categorized as non-rapidly fatal cases. One demonstrated many positive vessels, but the remaining 4 cases possessed only 1 or 2 positive vessels.

Analysis of the cases negative for the red pattern revealed that 2 populations lacked this pattern. One was the cases of hemorrhagic shock (n = 10) in the trauma group. The other was younger patients: all asphyxia patients under 3 years of age (n = 3) and 2 of the 3 young patients (12 and 29 years old) in the circulatory disease group.

### The correlation of tinctorial pattern with rapidity of death

**Figure 4:**
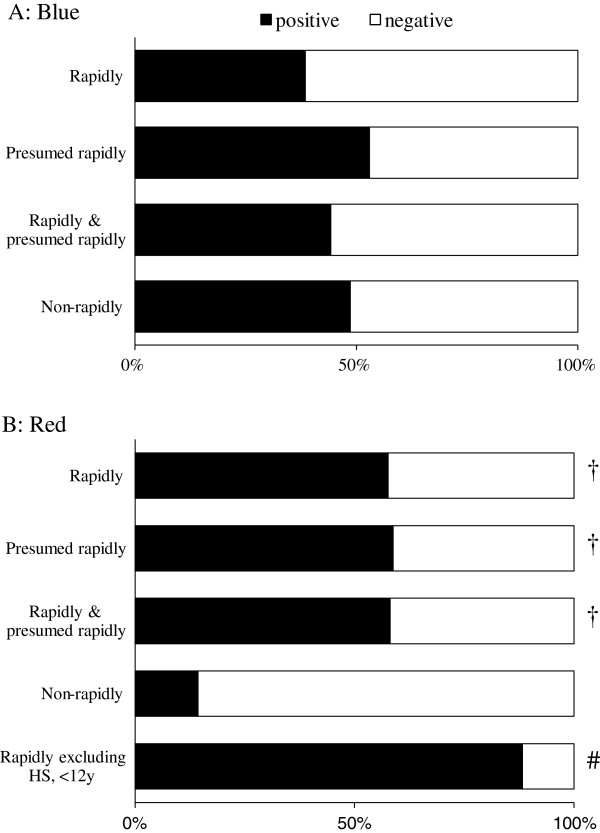
**The frequency of cases with (A) the blue pattern and (B) the red pattern in each group divided according to rapidity of death.** Rapidly indicates rapidly fatal cases; presumed rapidly, presumed rapidly fatal cases; rapidly & presumed rapidly, combination of rapidly and presumed rapidly fatal cases; non-rapidly, non-rapidly fatal cases; HS, hemorrhagic shock cases; <12 y, cases under 12 years of age; black box, positive cases; white box, negative cases. (A) The blue pattern did not significantly differ in appearance frequency across the categories. (B) Compared to non-rapidly fatal cases, the red pattern appeared with higher frequency in the rapidly and/or presumed rapidly cases, especially excluding hemorrhagic shock cases and those under 12 years of age (^†^*P* < 0.05, ^#^*P* < 0.01 vs. non-rapidly fatal death; ***χ***^2^ contingency test).

The previous analyses demonstrated different characteristics in the appearance of the blue and red hyaline. Blue hyaline was increasingly frequent with age and unrelated to cause of death, while red hyaline was unrelated to age and demonstrated different appearance frequencies according to cause of death. The groups with high frequency consisted of the circulatory diseases group and the drowning and asphyxia group, which generally have a tendency to progress rapidly. Indeed, 25 of 30 cases with red hyaline were categorized as rapidly fatal death or presumed rapidly fatal death, while only 18 of 48 cases without red hyaline were categorized into those 2 groups (*P <* 0.05, *χ*^2^ contingency test). Therefore, we hypothesize from these results that the tinctorial patterns may be applicable to assessing the rapidity of death. The appearance frequencies of the 2 patterns were evaluated in each of the following groups (Figure 4): rapidly fatal death, presumed rapidly fatal death, non-rapidly fatal death, and a combination of rapidly fatal death and presumed rapidly fatal death.

The blue pattern exhibited similar frequencies across the groups (Figure 4A): rapidly fatal death (38%, 10/26), presumed rapidly fatal death (53%, 9/17), combination of rapidly and presumed rapidly fatal death (44%, 19/43), and non-rapidly fatal death (49%, 17/35). The frequency in the rapidly, presumed rapidly, and combination of rapidly and presumed rapidly groups was not significantly higher compared to that of the non-rapidly fatal cases (*P* > 0.05, *χ*^2^ contingency test). By contrast, the red pattern exhibited different frequencies across the groups (Figure 4B). This pattern was found in only 14% (5/35) of non-rapidly fatal cases, but found in 58% (15/26), 59% (10/17), and 58% (25/43) of the rapidly, presumed rapidly, and combination of rapidly and presumed rapidly fatal cases, respectively. Statistical analysis revealed that the frequency of the red pattern in the rapidly, presumed rapidly, and combination of rapidly and presumed rapidly fatal cases was significantly higher than that in the non-rapidly fatal cases (*P* < 0.01).

Even among the rapidly fatal cases, 11 cases lacked the red pattern (Figure 4B, white box in top bar). A noticeable characteristic of these cases was that most cases (9/11, 82%) belonged to the 2 categories that contained no positive cases: hemorrhagic shock and children under 12 years of age. Thus, these 2 groups were deemed not to show the red pattern in any situation. When these 2 groups were excluded from the analysis, the appearance frequency of the red pattern rose to 88% (15/17) (Figure 4B, bottom bar).

To evaluate the applicability of the red pattern to assessing the rapidity of death, we calculated its sensitivity, specificity, positive predictive value, and negative predictive value in each of following groups (Table 2): rapidly fatal death, combination of rapidly fatal death with presumed rapidly fatal death, and rapidly fatal death excluding the cases with hemorrhagic shock and those under 12 years of age. Similar results were obtained for the former 2 groups in each index: sensitivity, 57.7% and 58.1%; specificity, both 85.7%; positive predictive value, 75.0% and 83.3%; and negative predictive value, 62.5% and 73.2%. When we calculated these indices in the rapidly fatal cases excluding hemorrhagic shock or under 12 years of age, sensitivity and negative predictive value rose to 88.2% and 92.6%, respectively.

**Table 2:** Diagnostic accuracy of red pattern for rapidity of the death

**Group**	**Sensitivity**	**Specificity**	**Positive predictive value**	**Negative predictive value**
**vs. non-rapidly**	**(95% CI)**	**(95% CI)**	**(95% CI)**	**(95% CI)**
**Rapidly**	57.7%	85.7%	75.0%	73.2%
	(0.369 to 0.766)	(0.697 to 0.952)	(0.509 to 0.913)	(0.571 to 0.858)
**Combination of Rapidly and Presumed-rapidly**	58.1%	85.7%	83.3%	62.5%
	(0.421 to 0.730)	(0.697 to 0.952)	(0.653 to 0.944)	(0.474 to 0.760)
**Rapidly excluded HS, < 12 yrs**	88.2%	83.3%	75.0%	92.6%
	(0.636 to 0.985)	(0.653 to 0.944)	(0.509 to 0.913)	(0.757 to 0.991)

*CI*, indicates confidence intervals; *HS*, hemorrhagic shock cases; <12 yrs, cases under 12 years of age.

## Discussion

The present study revealed that blue Azan-stained hyaline in splenic arterioles is increasingly frequent with age, and red Azan-stained hyaline is found frequently in cases of circulatory disease and drowning and asphyxia. Many previous studies have reported that the incidence of hyaline change increases with age [3-5,28], similar to the blue pattern shown in our study. Since those researchers used mainly periodic acid-Schiff (PAS) as a histochemical method to observe hyaline, their hyaline may have been stained blue if they had alternatively used the Azan stain. The blue color on Azan staining generally indicates that hyaline has formed gradually over prolonged periods [18-21], consistent with our observation of the blue pattern more frequently in older age groups in the present study. The association between this type of arteriolar hyaline and atherosclerotic change/consequences prevalent in elderly has been investigated previously [29-31]. Plesea et al. revealed that areas of hyaline material in the vascular wall are points of low resistance, and therefore possible site of wall rupture in the case of abrupt increase of blood pressure, resulting in hypertensive intracerebral hemorrhage [31].

The red color on Azan staining was unrelated to age and was found frequently among rapidly fatal cases such as the circulatory disease group and the drowning and asphyxia group. This color is principally reflected by fibrin contained within hyaline change [18-21]. The fibrin is generated due to activation of the coagulation cascade and can be formed in a short time. That is, the red pattern on Azan staining indicates the presence of fibrin in hyaline change, suggesting that the hyaline was recently generated. Therefore, the red pattern described in our study was likely generated just prior to the fatal accident, leading to its frequent appearance in the rapidly fatal cases. Unlike previous reports, this study was designed for forensic autopsy cases and contains many rapidly fatal cases. This characteristic of the study population may have led to the discovery of the new significance of the red pattern in autopsy.

Arteriolar hyaline change has been assumed to derive from plasma protein leakage across injured endothelial cells generated as a result of a general process, probably hemodynamic, e.g., hypertension, rather than as a consequence of any particular disease state [2,6,9,10,12]. This generation process is consistent with our observation of the red hyaline among all cause-of-death groups, although our results also seem to have an association with circulatory diseases. Feigin et al. investigated the significance of the hyaline colors of cerebral arteries using the tinctorial properties of the Azan stain as in our study [18]. These authors termed red hyaline “the fibrinoid form of hyaline” and reported that the form was most specifically related to hypertension and frequently observed in cerebral hemorrhage. The red pattern in our study is equivalent to Feigin’s “fibrinoid form” and was probably produced through endothelial damage due to rapid elevation of blood pressure induced by stress immediately prior to death. The central arteries and penicillar arterioles of the spleen were the vessels examined in this study. These vessels are among the terminal arterioles distributed throughout the body. However, we could not identify the same lesion in any other organs despite detailed microscopic examination. The distinctive vascular network of the spleen [26,27] may influence the formation of the red pattern*.* A further examination of the mechanism for the formation of the red pattern is beyond the scope of the present report, but will be of interest for future studies.

This study demonstrates that the red pattern of Azan staining may help pathologists determine the cause of death. Distinguishing hypothermia from acute coronary syndrome as the cause of death after cold exposure is occasionally difficult in practical situations because no established pathological findings of death from acute coronary syndrome within several hours are available. However, when splenic arteriolar hyaline shows the red pattern with Azan staining, we can infer that the cause of death is acute coronary syndrome rather than hypothermia, because the frequency of the red pattern is significantly higher in circulatory diseases compared to hypothermia (Figure 3, right column). Although the determination of the cause of death should be, of course, based on overall evidence, our results could provide a diagnostic clue in a challenging case to pathologists.

The presence of red pattern, although not very sensitive, was highly specific (85.7%) to rapidly fatal cases in this study. We examined a single splenic section from each patient, and the use of 2 or more sections could increase its sensitivity. However, examination of multiple sections is not a practical method. To increase the sensitivity, we propose that cases of hemorrhagic shock and those under 12 years of age be excluded from this assessment of the rapidity of death using the red pattern, because these cases did not show the pattern even when they presented a rapidly fatal course. When these cases were excluded from the evaluation of diagnostic accuracy of the red pattern for rapidity of death, each index increased, as shown in Table 2. The reason for these cases failing to demonstrate the pattern is not clear. However, the reduction of blood volume due to hemorrhage may interfere with a rapid elevation of blood pressure, and the mechanism of autoregulation in children may overcome hypertension [32].

The combination of the 2 colors shown in Figure 1A was excluded from detailed analyses in this study. The presence of this pattern did not significantly differ across age or cause-of-death groups (data not shown). This pattern is not useful in practical situations because it probably reflects a mixture of other patterns such as intermediate stages from the red to the blue pattern, in which materials from different stages coexist and the staining reactions are mixed. The limitations in our study are (1) a small number of cases from a single institution, (2) reliance on the presence of a witness or document for the determination of rapidity of death, and (3) no examination for the components of red Azan-stained hyaline. However, to our knowledge, this report is the first to estimate the applicability of splenic arteriolar hyaline for diagnosing the cause and rapidity of death.

## Conclusions

The estimation of hyaline deposits in the central artery and penicilli of the spleen, and specifically the red pattern, with Azan staining in adult cases without hemorrhage is useful for the diagnosis of rapidly fatal cases.

## Competing interests

The authors declare that they have no competing interests.

## Authors’ contributions

HK conceived of the study, carried out the case collection, concluded the cause of death, participated in the assessment of hyaline, and drafted the manuscript. MM and SM participated in study design and performed the statistical analysis. TI, CK, and HA participated in the autopsy and the histological studies. KT concluded the cause of death, participated in study design and coordination, and helped to draft the manuscript. All authors read and approved the final manuscript.

## References

[B1] Gamble CN The pathogenesis of hyaline arteriolosclerosis Am J Pathol 1986 122 410 420 2420184 PMC1888226

[B2] Kumar V, Abbas AK, Fausto N, Aster JC Robbins and cotran pathologic basis of disease 2010 8th Philadelphia, PA: Saunders Elsevir

[B3] McKinney B Hyaline arteriolosclerosis in the spleen: I. Methods of study and data from europeans Exp Mol Pathol 1962 1 275 287 10.1016/0014-4800(62)90024-2

[B4] Ball MJ Silver MD Influence of age and hypertension on splenic hyaline arterioscerosis Can Med Assoc J 1968 99 1239 1245 4178291 PMC1945617

[B5] Smith JP Hyaline arteriolosclerosis in spleen, pancreas and other viscera J Pathol Bacteriol 1956 72 643 656 10.1002/path.1700720231

[B6] Lindley RP Splenic arteriolar hyalin in children J Pathol 1986 148 321 325 10.1002/path.1711480408 2422337

[B7] Gupta RK Schuster R Christian WD A comparative immunohistochemical study of splenic arterial hyalinosis in health and disease Am J Pathol 1972 69 79 88 4117028 PMC2032774

[B8] Dustin P Bartman J Anatomo-clinical research on splenic hyalinosis in the child Rev Belg Pathol Med Exp 1961 28 152 170 13888716

[B9] Saracco SM Farhi DC Splenic pathology in thrombotic thrombocytopenic purpura Am J Surg Pathol 1990 14 223 229 10.1097/00000478-199003000-00003 2106272

[B10] Chang CS Li CY Cha SS Chronic idiopathic thrombocytopenic purpura. Splenic pathologic features and their clinical correlation Arch Pathol Lab Med 1993 117 981 985 8215839

[B11] Jellinek H Fibrinoid vascular changes showing the same morphologic pattern following induction by various experimental conditions Angiology 1967 18 547 555 10.1177/000331976701800902 6050802

[B12] Daneshbod K Liao KT Hyaline degeneration of splenic follicular arteries in infectious mononucleosis: Histochemical and electron microscopic studies Am J Clin Pathol 1973 59 473 479 4120872 10.1093/ajcp/59.4.473

[B13] Crawford T Woolf N Hyaline arteriolosclerosis in the spleen: an immuno-histochemical study J Pathol Bacteriol 1960 79 221 225 10.1002/path.1700790202 13812725

[B14] Cooper JH Haq BM Bagnell H Intrafollicular hyalinosis and arterial hyalinosis of the spleen: Histochemical and immunofluorescence studies J Pathol 1969 98 193 199 10.1002/path.1710980306 4187536

[B15] Krawczyński K Immunohistochemical study of arteriolar (simple) hyalinosis in spleen Am J Pathol 1971 62 253 264 4100062 PMC2047530

[B16] Hall CE Electron microscopy of fibrinogen and fibrin J Biol Chem 1949 179 857 864 18150018

[B17] Cohen C Weisel JW Phillips GN Stauffacher CV Fillers JP Daub E The structure of fibrinogen and fibrin: I. Electron microscopy and x-ray crystallography of fibrinogen Ann N Y Acad Sci 1983 408 194 213 10.1111/j.1749-6632.1983.tb23245.x 6575684

[B18] Feigin I Prose P Hypertensive fibrinoid arteritis of the brain and gross cerebral hemorrhage: a form of “Hyalinosis” AMA Arch Neurol 1959 1 98 110 10.1001/archneur.1959.03840010100012 13821920

[B19] Lendrum AC Fraser DS Slidders W Henderson R Studies on the character and staining of fibrin J Clin Pathol 1962 15 401 413 10.1136/jcp.15.5.401 13929601 PMC480427

[B20] Heidenhain M Uber die mallorysche bindegewebsfarbung mit karmin und azokarmin als vorfarben Zeitschrift fur wissenschafthche Mikroskopie und fur mikroskopische Technik 1915 32 361 372

[B21] Kiernan JA Histological and histochemical methods theory and practice 4th edition, ed 2008 4th Oxfordshire: Scion Publishing Limited

[B22] DiMaio VJ DiMaio D Forensic pathology second edition 2001 2nd Florida: CRC Press

[B23] Weber MA Pryce JW Ashworth MT Malone M Sebire NJ Histological examination in sudden unexpected death in infancy: Evidence base for histological sampling J Clin Pathol 2012 65 58 63 10.1136/jclinpath-2011-200224 21965829

[B24] Oliva A Brugada R D’Aloja E Boschi I Partemi S Brugada J Pascali VL State of the art in forensic investigation of sudden cardiac death Am J Forensic Med Pathol 2011 32 1 16 10.1097/PAF.0b013e3181c2dc96 20083991

[B25] Basso C Burke M Fornes P Gallagher PJ de Gouveia RH Sheppard M Thiene G van der Wal A Pathology AfEC: Guidelines for autopsy investigation of sudden cardiac death Virchows Arch 2008 452 11 18 10.1007/s00428-007-0505-5 17952460

[B26] Snook T The origin of the follicular capillaries in the human spleen Am J Anat 1975 144 113 117 10.1002/aja.1001440107 1166852

[B27] Schmidt EE MacDonald IC Groom AC Microcirculatory pathways in normal human spleen, demonstrated by scanning electron microscopy of corrosion casts A J Anat 1988 181 253 266 10.1002/aja.1001810304 3364384

[B28] Moritz AR Oldt MR Arteriolar sclerosis in hypertensive and non-hypertensive individuals Am J Pathol 1937 13 679 728 19970344 PMC1911138

[B29] Lakhan S Harle L Cardiac fibrosis in the elderly, normotensive athlete: Case report and review of the literature Diagn Pathol 2008 3 12 10.1186/1746-1596-3-12 18353184 PMC2277381

[B30] Nakamura S Ishibashi-Ueda H Suzuki C Nakata H Yoshihara F Nakahama H Kawano Y Renal artery stenosis and renal parenchymal damage in patients with abdominal aortic aneurysm proven by autopsy Kidney Blood Press Res 2009 32 11 16 10.1159/000197865 19176973

[B31] Plesea IE Camenita A Georgescu CC Enache SD Zaharia B Georgescu CV Tenovici M Study of cerebral vascular structures in hypertensive intracerebral haemorrhage Rom J Morphol Embryol 2005 46 249 256 16444313

[B32] Gorm G Autoregulation of cerebral blood flow in newborn babies Early Hum Dev 2005 81 423 428 10.1016/j.earlhumdev.2005.03.005 15935919

